# γ And β Band Oscillation in Working Memory Given Sequential or Concurrent Multiple Items: A Spiking Network Model

**DOI:** 10.1523/ENEURO.0373-22.2023

**Published:** 2023-11-07

**Authors:** Shukuo Zhao, Jinpu Zhou, Yongwen Zhang, Da-Hui Wang

**Affiliations:** 1 School of Systems Science, Beijing Normal University, Beijing 100875, China; 2 State Key Laboratory of Cognitive Neuroscience and Learning, Beijing Normal University, Beijing 100875, China; 3 Beijing Key Laboratory of Brain Imaging and Connectomics, Beijing Normal University, Beijing 100875, China

**Keywords:** oscillation, sequential/concurrent items, spiking network, working memory

## Abstract

Working memory (WM) can maintain sequential and concurrent information, and the load enhances the γ band oscillation during the delay period. To provide a unified account for these phenomena in working memory, we investigated a continuous network model consisting of pyramidal cells, high-threshold fast-spiking interneurons (FS), and low-threshold nonfast-spiking interneurons (nFS) for working memory of sequential and concurrent directional cues. Our model exhibits the γ (30–100 Hz) and β (10–30 Hz) band oscillation during the retention of both concurrent cues and sequential cues. We found that the β oscillation results from the interaction between pyramidal cells and nFS, whereas the γ oscillation emerges from the interaction between pyramidal cells and FS because of the strong excitation elicited by cue presentation, shedding light on the mechanism underlying the enhancement of γ power in many cognitive executions.

## Visual Abstract

**Figure:**
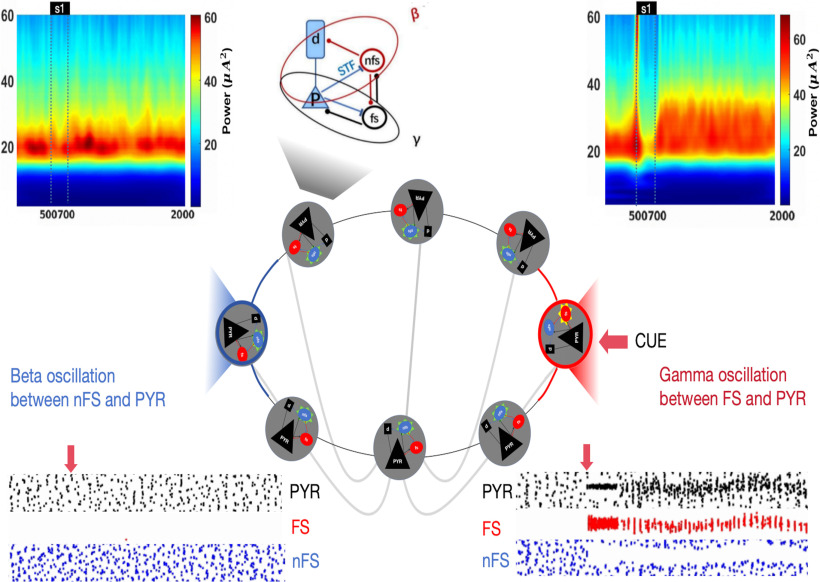

## Significance Statement

We constructed a spiking network to perform working memory (WM) tasks with sequentially or concurrently presented items. The model exhibits the coexistence of β (10–30 Hz) and γ (30–100 Hz) band oscillations during the delay period. We found that γ and β band oscillations recruit separate neural circuits. The low-threshold nonfast-spiking (nFS) neurons are involved in the β band oscillation, whereas the high-threshold fast-spiking (FS) neurons are involved in the γ band oscillation. Our results shed light on the well-known phenomenon that cognitive tasks enhance γ band oscillations.

## Introduction

Working memory (WM), the ability to actively maintain and manipulate information in the absence of stimuli, plays a crucial role in cognitive function and executive control of behavior (Baddeley, 2003). The information maintained in WM can enter the brain concurrently or sequentially, meaning that WM cannot only hold multiple pieces of information arriving simultaneously as in visuospatial WM tasks (Bays and Husain, 2008; Zhang and Luck, 2008; Gorgoraptis et al., 2011), but also information presented sequentially, as in visual (Gorgoraptis et al., 2011) or speech processing (Cowan, 2010). The neural implementation of information maintenance is not well understood. The oscillatory model proposes that the information of one item is represented in WM by the reactivation of neurons in the γ cycle within nested γ-theta oscillations, mediated by a slow after-depolarization (ADP) with a time constant that should match the theta oscillation (Lisman and Idiart, 1995; Jensen and Lisman, 1998). The dynamic model posits that memory is maintained by item-specific patterns of synaptic plasticity and that neurons exhibit a nonstationary and short-lived attractor activity, in which only one memory representation can be active at a time, but successive reactivations of neuronal pools memorize different items (Mongillo et al., 2008; Lundqvist et al., 2016, 2018; Mi et al., 2017). The persistent activity model asserts that persistent activity of neurons induced by *N*-methyl-D-aspartate receptor (NMDAR)-mediated recurrent synaptic current encodes the corresponding items during the delay period (Compte et al., 2000; Edin et al., 2009; Wei et al., 2012). The ability of WM to maintain concurrent and sequential information challenges these models. On the one hand, the oscillatory model and dynamic models can use oscillatory activity to maintain sequential information, but it is difficult for them to represent concurrent information (Constantinidis et al., 2018); on the other hand, the persistent models can manipulate concurrent information but cannot address sequential stimuli and often focus on the persistent activity without oscillation. Experiments have shown that γ band oscillations are involved in WM maintenance, in particular, that WM load enhances the γ band oscillations but suppresses the alpha band oscillations. Here, we aim to construct a biophysically plausible network model to implement the storage of concurrent and sequential information and to investigate how WM load alters oscillations in the network.

The biological brain consists of microcircuits with three types of neurons that play key roles in generating oscillations: pyramidal cells, low-threshold nonfast-spiking (nFS), and high-threshold neuronal FS (Chen et al., 2017; Veit et al., 2017; Domhof and Tiesinga, 2021; Hahn et al., 2022). The pyramidal cells are excitatory, while the FS and nFS are GABAergic inhibitory. Pyramidal cells synapse on nFS and FS. In turn, nFS neurons synapse back onto the dendrites of the pyramidal cell, while FS neurons synapse back onto the soma of the pyramidal cell, creating a dynamic feedback loop that regulates excitatory and inhibitory activities. FS and nFS also inhibit each other, resulting in a competitive relationship. At rest, the nFS is more active, inhibiting the FS and forming a β oscillation with the pyramidal cells. When a perceptual stimulus is presented, the FS cells are activated, inhibiting the nFS cells and creating a γ oscillation with the pyramidal cells (Chen et al., 2017; Hahn et al., 2022). Given the importance of oscillations in higher cognitive functions, we aim to explore the role of oscillations and microcircuitry in working memory.

We proposed a spiking neural network to implement WM with concurrent and sequential directional information (Gorgoraptis et al., 2011). The network consists of two-compartment pyramidal cells, nFS, and FS. These cells and compartments are interconnected as in the biological brain. The cells are uniformly arranged in a ring according to their preferred direction (Compte et al., 2000). When activated, pyramidal cells activate pyramidal cells with similar preferences as well as nearby FS cells and nFS cells on the ring. FS and nFS inhibit neighboring neurons on the ring. We found that, regardless of whether the directions are presented concurrently or sequentially, the network elicits corresponding localized activities that persist throughout the delay period. The persistent activity suggests that the model can successfully maintain multiple stimuli presented simultaneously or sequentially in working memory. Furthermore, before cue presentation, the interaction between pyramidal cells and nFS dominates the activity of the network and causes a low-band oscillation (10–30 Hz); the cue presentation induces strong excitation and recruits FS into the network, and the interaction between pyramidal cells and FS dominates the activity of the network, enhancing the γ oscillation (35–100 Hz), which persists throughout the delay period.

## Materials and Methods

### Model architecture

Our model has 4096 excitatory pyramidal cells, 512 FS cells, and 512 nFS cells. We have two reasons to choose the number of neurons. First, the ratio of the number of excitatory neurons over that of inhibitory neurons is ∼4:1 (Braitenberg and Schutz, 1991). Thus, we chose 4906 pyramidal neurons and 1024 inhibitory interneurons (FS + nFS, 1024). Second, a network size of $2^{n}$ neurons is desired for the fast Fourier transform (FFT) which recursively divides the input data into smaller subsets, and conducts Fourier transform computations on subsets. These neurons are evenly distributed in a ring configuration and are connected by AMPA, NMDA, and GABA synapses, forming an interconnected spiking network. According to our model, the strength of connections between neurons and the angular differences in their distribution across the ring configuration follows a Gaussian distribution. Neurons that are closer in angle on the ring have stronger connections. The footprint of the connections can be described as Equation 1:

(1) $$\{\begin{array}{c} \text{W}\left({\text{θ}}_{\text{i}}-{\text{θ}}_{\text{j}}\right)={\text{J}}^{-}+\left({\text{J}}^{+}-{\text{J}}^{-}\right)\exp\left(-\frac{{\left({\text{θ}}_{\text{i}}-{\text{θ}}_{\text{j}}\right)}^{2}}{2{\text{σ}}^{2}}\right){\text{θ}}_{\text{i}}\in \text{E}\\ \text{W}\left({\text{θ}}_{\text{i}}-{\text{θ}}_{\text{j}}\right)=\exp\left(-\frac{{\left({\text{θ}}_{\text{i}}-{\text{θ}}_{\text{j}}\right)}^{2}}{4{\text{π}}^{2}{\text{σ}}^{2}}\right){\text{θ}}_{\text{i}}\in \text{l} \end{array}.$$

${\text{J}}_{\text{s}\to \text{s}}^{+}=6.5$, ${\text{ J}}_{\text{s}\to \text{fs}}^{+}=35$, ${\text{ J}}_{\text{s}\to \text{nfs}}^{+}=70$. By normalizing the footprint $\frac{1}{360}\overset{360}{\underset{0}{\int }}\text{W}\left({\text{θ}}_{\text{i}}-{\text{θ}}_{\text{j}}\right){\text{dθ}}_{\text{j}}=1$, we obtain ${\text{J}}^{-}$ in Equation 1. The standard deviations of the Gaussian distributions for connectivity footprint in our model were as follows:

- From pyramidal cells soma to pyramidal cells soma ( ${\text{σ}}_{\text{s}\to \text{s}}$): 12.76°

- From pyramidal cells soma to FS cell ( ${\text{σ}}_{\text{s}\to \text{fs}}$): 7.05°

- From pyramidal cells soma to nFS cell ( ${\text{σ}}_{\text{s}\to \text{nfs}}$): 1.41°

- From FS cell to pyramidal cells soma ( ${\text{σ}}_{\text{fs}\to \text{s}}$): 8.46°

- From FS cell to nFS cell ( ${\text{σ}}_{\text{fs}\to \text{nfs}}$): 1.41°

- From nFS cell to pyramidal cells dendrite ( ${\text{σ}}_{\text{nfs}\to \text{d}}$): 5.02°

The value of parameters ${\text{J}}_{\text{s}\to \text{s}}^{+}$ and ${\text{σ}}_{\text{s}\to \text{s}}$ used in our study were adapted from Compte et al. (2000) and Wei et al. (2012). We further introduced structured excitatory projections from pyramidal cells to FS and nFS and inhibitory projections from FS to pyramidal soma and nFS cells or from nFS to pyramidal dendrite, referencing local circuit properties described by Wang et al. (2004) and Wang and Yang (2018). FS belongs to the large basket cell that projects to broader region, while nFS has narrow dendritic and axonal arbors and serves local inhibition. Generally, excitatory neurons project to a larger radius (around 200 μmphysiologically; as shown by Fitzpatrick et al., 1985) compared with inhibitory interneurons (around 100 μm; as demonstrated by Spiro et al., 1999) based on biological experiments. Nevertheless, the lateral innervation width of interneurons may be similar to that of excitatory neurons. It is because excitatory neurons project to a larger radius, activating inhibitory neurons within that radius to locally innervate inhibition (as assumed by Compte et al). The synaptic weights were calculated by the production of footprint and the maximum conductance as follows:

(2) $${\text{g}}_{\text{ij}}=\text{W}\left({\text{θ}}_{\text{i}}-{\text{θ}}_{\text{j}}\right){\text{G}}_{\text{syn}}.$$

**Figure 1.**
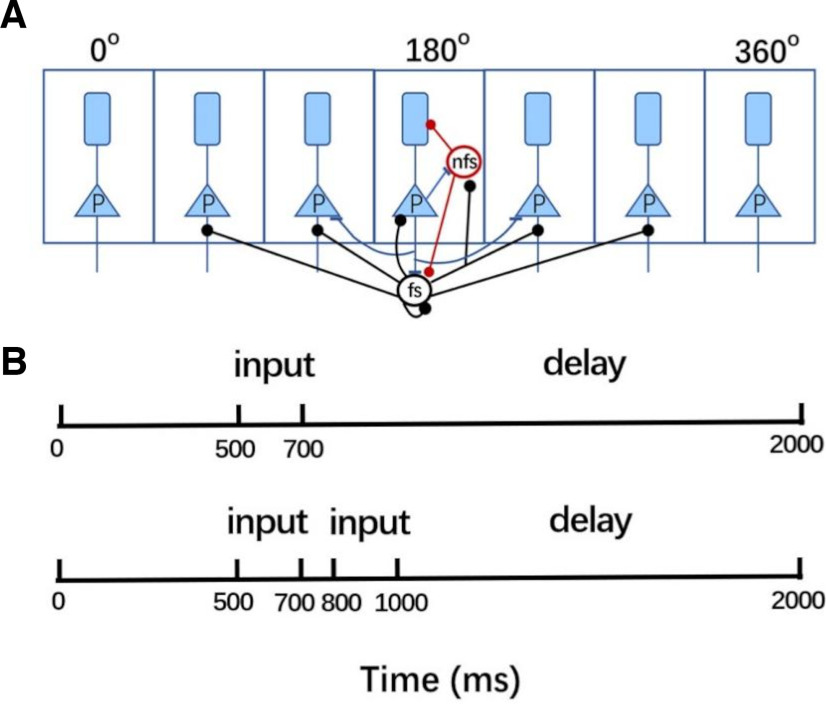
Model structure and simulation protocol. ***A***, The network consists of two-compartment pyramidal cells, fast-spiking (FS), and nonfast-spiking (nFS) interneurons. Pyramidal and inhibitory cells are uniformly placed on a ring and labeled by their preferred directions. ***B***, Simulation protocol. In concurrent-cue tasks, an array of cues is presented to the network from 500 to 700 ms, followed by a delay period of 1300 ms (upper panel). In sequential-cue tasks, cues were presented one after another with an interval of 100 ms, and each cue lasted for 200 ms.

${\text{The G}}_{\text{syn}}$ parameters for these connections are specified in Table 1 (Compte et al., 2000; Wei et al., 2012). To simplify the calculations, we have assumed that the remaining inhibitory connections in our model (e.g., inhibitory connection from nFS to FS or recurrent connection from FS to FS) follow a uniform distribution (a simple diagram is shown in Fig. 1).

**Table 1:** G_syn_ Parameters for soma, dendrite, FS, and nFS synaptic connections

		Presynaptic neurons			
		Pyr-soma		FS	nFS
		AMPA	NMDA	GABA	GABA
Postsynaptic neurons	Pyr-soma	0.0001125	0.0000750	0.0003420	-
Pyr-dendrite	-	-	-	0.0018210	
FS	0.0001600	0.0000600	0.0000600	0.0000900	
nFS	0.0002400	0.0002400	0.0105000	-	

This table specifies the parameters used for calculating the AMPA, NMDA, and GABA synaptic connections between soma, dendrite, FS, and nFS [adapted from Compte et al. (2000) and Wei et al. (2012)].

### Neuron models

Pyramidal cells and inhibitory cells follow the leaky integrate-and-fire model (Tuckwell, 1988). The neurons generate spikes when their membrane potentials reach threshold ( ${\text{V}}_{\text{th}}=-50\text{mV}$.) And their membrane potential is reset to ${\text{V}}_{\text{reset}}=-60\text{mV}$ immediately after a spike for ${\text{τ}}_{\text{s}}=2\text{ms}$; ${\text{τ}}_{\text{d}}=2\text{ms}$; ${\text{τ}}_{\text{fs}}=$1ms; ${\text{τ}}_{\text{nfs}}=$1ms as refractory periods. The Pyramidal cells have two compartments (Wang, 1998): soma(s) and dendrite(d). The membrane potential of the somatic compartment (Vs) and dendrite compartment (Vd) of pyramidal cells obey Equations 3 and 4:

(3) $${\text{C}}_{\text{m}}\frac{{\text{dV}}_{\text{s}}}{\text{dt}}=-{\text{I}}_{\text{Ca}}-{\text{g}}_{\text{sL}}\left({\text{V}}_{\text{s}}-{\text{V}}_{0}\right)-\frac{{\text{gc}}_{1}\left({\text{V}}_{\text{s}}-{\text{V}}_{\text{d}}\right)}{{\text{p}}_{1}}-{\text{I}}_{\text{syn}}\text{,}$$

(4) $${\text{C}}_{\text{m}}\frac{{\text{dV}}_{\text{d}}}{\text{dt}}=-{\text{g}}_{\text{dL}}\left({\text{V}}_{\text{d}}-{\text{V}}_{0}\right)-\frac{{\text{gc}}_{1}\left({\text{V}}_{\text{d}}-{\text{V}}_{\text{s}}\right)}{{\text{p}}_{2}}-{\text{I}}_{\text{syn}}.$$

${\text{V}}_{0}=-70\text{ mV}$, ${\text{ C}}_{\text{m}}=0.5\text{ nF}$, ${\text{g}}_{\text{sL}}=0.025nS$, ${\text{g}}_{\text{dL}}=0.025nS$, ${\text{gc}}_{1}=0.25nS$ (Troyer and Miller, 1997; Wei et al., 2012). The parameters p1 and p2 characterize the difference of current intensity resulting from the identical current in soma and dendrite because of the difference in membrane surface area. Specifically, p1 represents the ratio of somatic area to the total neuronal area, and p2 represents the ratio of dendritic area to the total area (Wang, 1998). In the current model, p1 is set to 0.6, and p2 is set to 1-p1, which is 0.4. FS and nFS cells contain single compartments, and their membrane potentials obey Equations 5 and 6. Please note that when the neuron (soma) fires, the membrane potential of the dendrite component is not reset to −60 mV. The dendrite component generates its own spikes and resets the membrane potential after each spike.

(5) $${\text{C}}_{\text{m}\_\text{fs}}\frac{{\text{dV}}_{\text{fs}}}{\text{dt}}=-{\text{g}}_{\text{fsL}}\left({\text{V}}_{\text{fs}}-{\text{V}}_{\text{fs}0}\right)-{\text{I}}_{\text{syn}}$$

(6) $${\text{C}}_{\text{m}\_\text{pv}}\frac{{\text{dV}}_{\text{nfs}}}{\text{dt}}=-{\text{I}}_{\text{Ca}}-{\text{g}}_{\text{nfsL}}\left({\text{V}}_{\text{nfs}}-{\text{V}}_{\text{nfs}0}\right)-{\text{I}}_{\text{syn}}.$$

${\text{C}}_{\text{m}\_\text{fs}}=0.2\text{ nF},{\text{C}}_{\text{m}\_\text{nfs}}=0.8\text{ nF},{\text{ g}}_{\text{fsL}}=0.020nS,{\text{g}}_{\text{nfsL}}=0.016nS,{\text{V}}_{\text{fs}0}=-86\text{ mV}$, ${\text{V}}_{\text{nfs}0}=-76\text{ mV}$ (Troyer and Miller, 1997; Wei, 2012). The term “Isyn” represents the total synaptic current. The ion current ${\text{I}}_{\text{Ca}}$ in Equations 3 and 6 represents a high-threshold calcium current following Equations 7 and 8 (Wang, 1998):

(7) $${\text{I}}_{\text{Ca}}={\text{g}}_{\text{Ca}}m_{\infty }^{2}(\text{V})(\text{V}-{\text{V}}_{\text{Ca}})$$

(8) $$m_{\infty }^{2}\left(\text{V}\right)=\frac{1}{1+e^{-(\text{V}+20)/9}}.$$

gCa−s=0.0015 nS, gCa−nfs=0.001 nS. V stands for membrane potential (equals ${\text{V}}_{\text{s}}$ or ${\text{V}}_{\text{nfs}}$).

### Synapse models

Synaptic currents are mediated by AMPA, NMDA, and GABA transmissions. Three types of synaptic currents follow the equations:

(9) $${\text{I}}_{\text{i},\text{AMPA}}=\left({\text{V}}_{\text{i}}-{\text{V}}_{\text{AMPA}}\right)\underset{\text{j}}{\sum }{\text{g}}_{\text{ji},\text{AMPA}}{\text{s}}_{\text{j},\text{AMPA}}$$

(10) $${\text{I}}_{\text{i},\text{NMDA}}=\left({\text{V}}_{\text{i}}-{\text{V}}_{\text{NMDA}}\right)\underset{\text{j}}{\sum }\frac{{\text{g}}_{\text{ji},\text{NMDA}}{\text{s}}_{\text{j},\text{NMDA}}}{1+\left[{\text{Mg}}^{2+}\right]\text{exp}\left(-0.026{\text{V}}_{\text{i}}/3.57\right)}$$

(11) $${\text{I}}_{\text{i},\text{GABA}}=\left({\text{V}}_{\text{i}}-{\text{V}}_{\text{GABA}}\right)\underset{\text{j}}{\sum }{\text{g}}_{\text{ji},\text{GABA}}{\text{s}}_{\text{j},\text{GABA}}.$$

The conductance “g” in our calculations primarily follows the Equation 2, ${\text{V}}_{\text{AMPA}}$= ${\text{V}}_{\text{NMDA}}=0\text{mV}$, ${\text{V}}_{\text{GABA}}=-70\text{mV}$, [Mg^2+^] in Equation 10 equals 1 mm (Jahr and Stevens, 1990). The gating variable s for AMPA or GABA ( ${\text{s}}_{\text{j},\text{AMPA}}$ and ${\text{s}}_{\text{j},\text{GABA}}$ in Eqs. 9, 11) follows the dynamic in Equation 12, and the dynamic of s ( ${\text{s}}_{\text{j},\text{NMDA}}$) for NMDA was modelled as Equations 13 and 14:

(12) $$\frac{\text{ds}\left(\text{t}\right)}{\text{dt}}=-\frac{\text{s}\left(\text{t}\right)}{{\text{τ}}_{\text{s}}}+\underset{\text{k}}{\sum }\text{δ}\left(\text{t}-{\text{t}}_{\text{k}}\right)$$

(13) $$\frac{\text{ds}\left(\text{t}\right)}{\text{dt}}=-\frac{\text{s}\left(\text{t}\right)}{{\text{τ}}_{\text{s},\text{NMDA}}}+{\text{α}}_{\text{s}}\text{x}\left(\text{t}\right)\left(1-\text{s}\left(\text{t}\right)\right)$$

(14) $$\frac{\text{dx}\left(\text{t}\right)}{\text{dt}}=-\frac{\text{x}\left(\text{t}\right)}{{\text{τ}}_{\text{x},\text{NMDA}}}+\underset{\text{k}}{\sum }\text{δ}\left(\text{t}-{\text{t}}_{\text{k}}\right).$$

${\text{t}}_{\text{k}}$ is the spike sequence of the neuron. The rising time constant for NMDA ( ${\text{τ}}_{\text{x},\text{NMDA}}$) is 2 ms, and the decay time constant ${\text{τ}}_{\text{s}}$ is 2 ms, 10 ms, and 100 ms for AMPA, GABA, and NMDA, respectively (Wei, 2012).

Synapses from Pyrs to nFS mediated by NMDAR were facilitated in short term and follow the equation:

(15) $$\frac{\text{du}}{\text{dt}}=\frac{\text{U}-\text{u}}{{\text{τ}}_{\text{f},\text{NMDA}}}+\text{k}\left(1-\text{u}\right)\text{δ}\left(\text{t}-{\text{t}}_{\text{k}}\right).$$

U is set to 0.4 in our simulation (Mongillo et al., 2008).

### Simulation protocol

We simulated two types of WM tasks: concurrent-cue tasks (CCTs) and sequential-cue tasks (SCTs), similar to the experiments in (Gorgoraptis et al., 2011). In CCTs, cues are presented to neurons concurrently; each pyramidal cell indexed by θ received a current ${\text{I}}_{\text{ext}}\left(\text{θ}\right)$ during the concurrent cue presentation period from 500 to 700 ms (Fig. 1*B*).

(16) $${\text{I}}_{\text{ext}}\left(\text{θ}\right)=\overset{n}{\underset{k=1}{\sum }}\frac{{\text{I}}_{0}}{\sqrt{2\pi }{\text{σ}}_{s}}\text{exp}\left(-\frac{{\left(\text{θ}-{\text{θ}}_{\text{in},\text{k}}\right)}^{2}}{2{\text{σ}}_{s}^{2}}\right),$$

where ${\text{θ}}_{\text{in},\text{k}}$ is the kth direction and n is the number of directions in the cue array. ${\text{I}}_{0}=0.5\text{ nA},\sigma_{s}=2$ degree.

In SCTs, pyramidal cells received multiple currents sequentially. Each current can be formulated as:

(17) $${\text{I}}_{\text{ext}}\left(\text{θ}\right)=\frac{{\text{I}}_{0}}{\sqrt{2\pi }{\text{σ}}_{s}}\text{exp}\left(-\frac{{\left(\text{θ}-{\text{θ}}_{\text{in},\text{k}}\right)}^{2}}{2{\text{σ}}_{s}^{2}}\right).$$

The kth current is applied at 500+300(k−1) to 700+300(k−1) ms (Fig. 1*B*) during the presentation of the kth cue (Wei et al., 2012).

### Noise implementation

Noise is applied to pyramidal cells and inhibitory cells via AMPA synapses:

(18) $${\text{I}}_{\text{i},\text{noise}}=\left({\text{V}}_{\text{i}}-{\text{V}}_{\text{AMPA}}\right){\text{g}}_{\text{noise}}{\text{s}}_{\text{noise}-\text{i}}$$

gnoise−pyr=0.0039 nS, gnoise−pv=0.0019 nS, gnoise−cb=0.0022 nS, and ${\text{s}}_{\text{noise}-\text{i}}$ follows:

(19) $$\frac{\text{ds}\left(\text{t}\right)}{\text{dt}}=-\frac{\text{s}\left(\text{t}\right)}{{\text{τ}}_{\text{s}}}+\eta (\text{t})$$

${\text{τ}}_{\text{s}}$ = 2 ms, and η(t) is a Poisson process.

(20) $$\text{p}(\text{η}(\text{t})=\text{k})=\frac{{\text{e}}^{-\text{λ*dt}}{\left(\text{λ * dt}\right)}^{\text{k}}}{\text{k}!},\text{k}=0,1...$$

where λ is the arrival rate of noise. We set λ = 1 kHz, which is equivalent to 1000 synaptic input at 1 Hz (Wei et al., 2012).

### Starting parameters

To establish a more realistic and stable initial condition, we conducted a recording procedure to obtain a consistent value of multiple variables for each type of neurons. The following variables were used: the membrane potential (“ ${\text{V}}_{\text{m}}$”), the gating variables (“s” and “x”) for synapses, the time of the last firing activity (“LastTimeEachFired” minus 250 ms), and the AMPA synaptic current (“I_AMPA”) for the pyramidal soma (over a period of 10 ms), and these recorded values were then used as initial conditions for the algorithms. Initially, we performed a model run with constant initialization values of −51 mV for the membrane potential and 1e-34 for the “s” parameter without these recordings.

### Numeric integration and code accessibility

The model was programmed in MATLAB code and the gating variables were integrated using a second-order Runge–Kutta (RK2) algorithm.

(21) $$x_{n+1}=x_{n}+\frac{1}{2}dt(K_{1}+K_{2})$$

(22) $$K_{1}=f(x_{n},t_{n})$$

(23) $$K_{2}=f(x_{n}+\text{dt}K_{1},t_{n}+dt).$$

The membrane potential is integrated with Euler scheme as Equations 3–6. This different choice in the integration method is because changes in gating variables during neuronal firing are more complex and usually nonlinear, whereas changes in the subthreshold membrane potential are approximately linear. We conducted a convergence test on two neurons with AMPA connections and provided a 2-mV constant stimulus to the presynaptic neuron for 2000 ms to examine the difference of spike time of postsynaptic neuron between Euler-RK2 and RK2 methods. We found that the spike time errors generated by the two methods are almost negligible for sufficiently small dt (such as 0.02, as in the experiments). By variation of the timesteps, we found the convergence order of integration method $\text{R}(=\frac{log_{2}||e_{new}||-log_{2}||e_{old}||}{log_{2}||\Delta t_{new}||-log_{2}||\Delta t_{old}||})$ equals 2.95, indicating that the error decays to 0.129 of its original error if the timestep is decreased by half.

This approach reduces the computational cost without compromising accuracy. The code/software described in the paper is freely available online at https://github.com/scientific-lab/Gamma-and-Beta-Band-Oscillation-in-Working-Memory. We performed our research using GPU(K80) with Ubuntu 18.04.6 LTS, Microsoft Surface Pro 5 with Windows 10 operating system, and MacBook Pro 11,1 with Windows 10 operating system.

### Data analysis

#### Firing rate calculation

**Figure 2.**
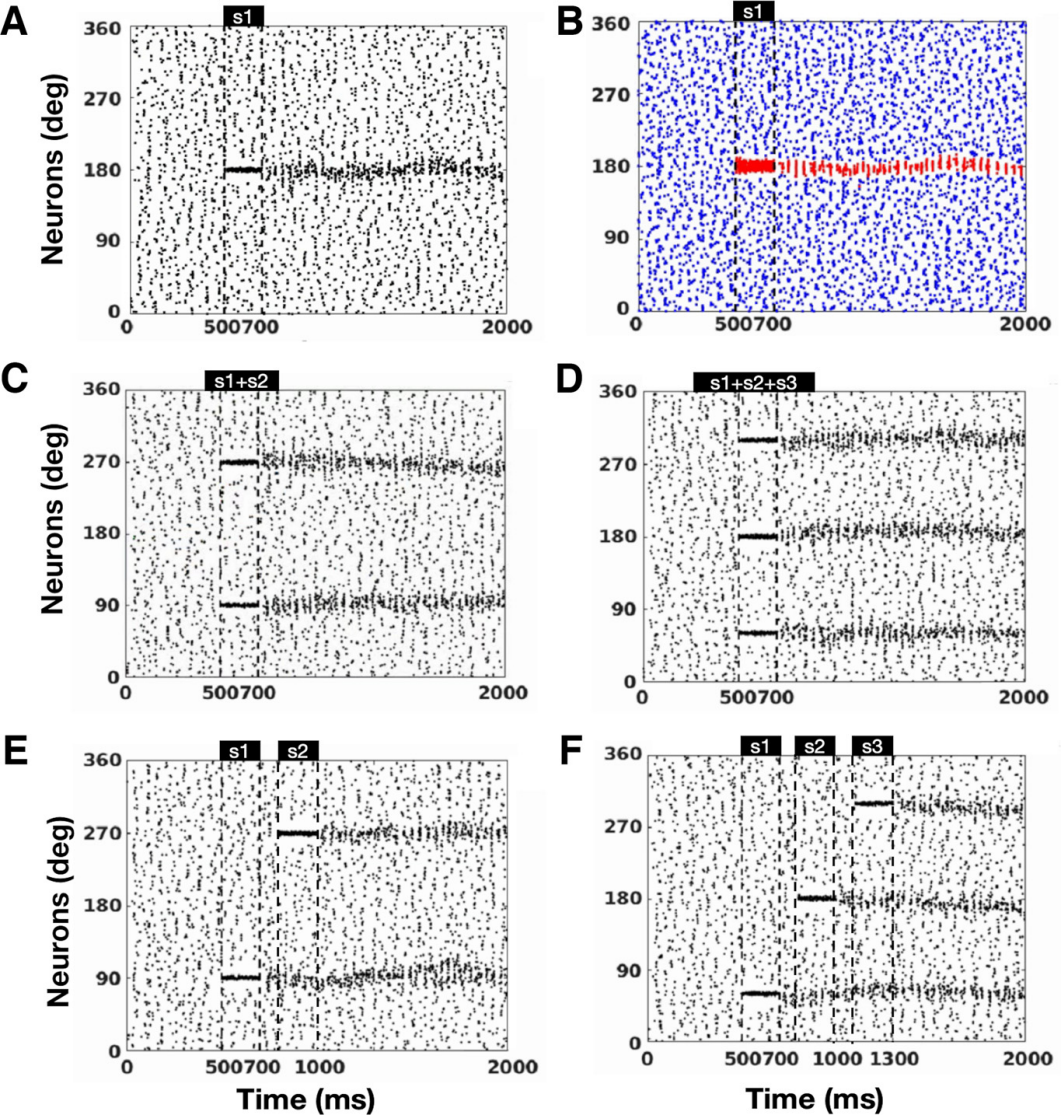
Raster plots of spiking of neurons in the network. ***A***, Raster plots of spiking of pyramidal cells given one cue. ***B***, Raster plots of spiking of low-threshold nFS (blue) and high-threshold FS (red) given one cue. ***C***, Two direction cues S1+S2 were concurrently presented to the network during the cue period (500–700 ms). ***D***, Three directional cues S1+S2+S3 were concurrently presented to the network during the cue period. ***E***, Two directional cues (S1 and S2) were sequentially presented to the network. ***F***, Three directional cues (S1, S2, and S3) were sequentially presented to the network. The horizontal axis is the time in milliseconds, and the vertical axis is the preferred direction of the pyramidal cell. s1-s3 represent the indices of the cue, and the dotted lines denote the cue presented interval(s). Please note that these symbols have the same interpretations in the following figures.

We recorded the firing activities of neurons (see Fig. 2) and counted the spikes of a certain type of neuron along the time in a single trial and approximated the firing rate by applying a one-dimension filter with a window size of 4 ms to the spike train.

#### Local field potential and spectrograms

**Figure 3.**
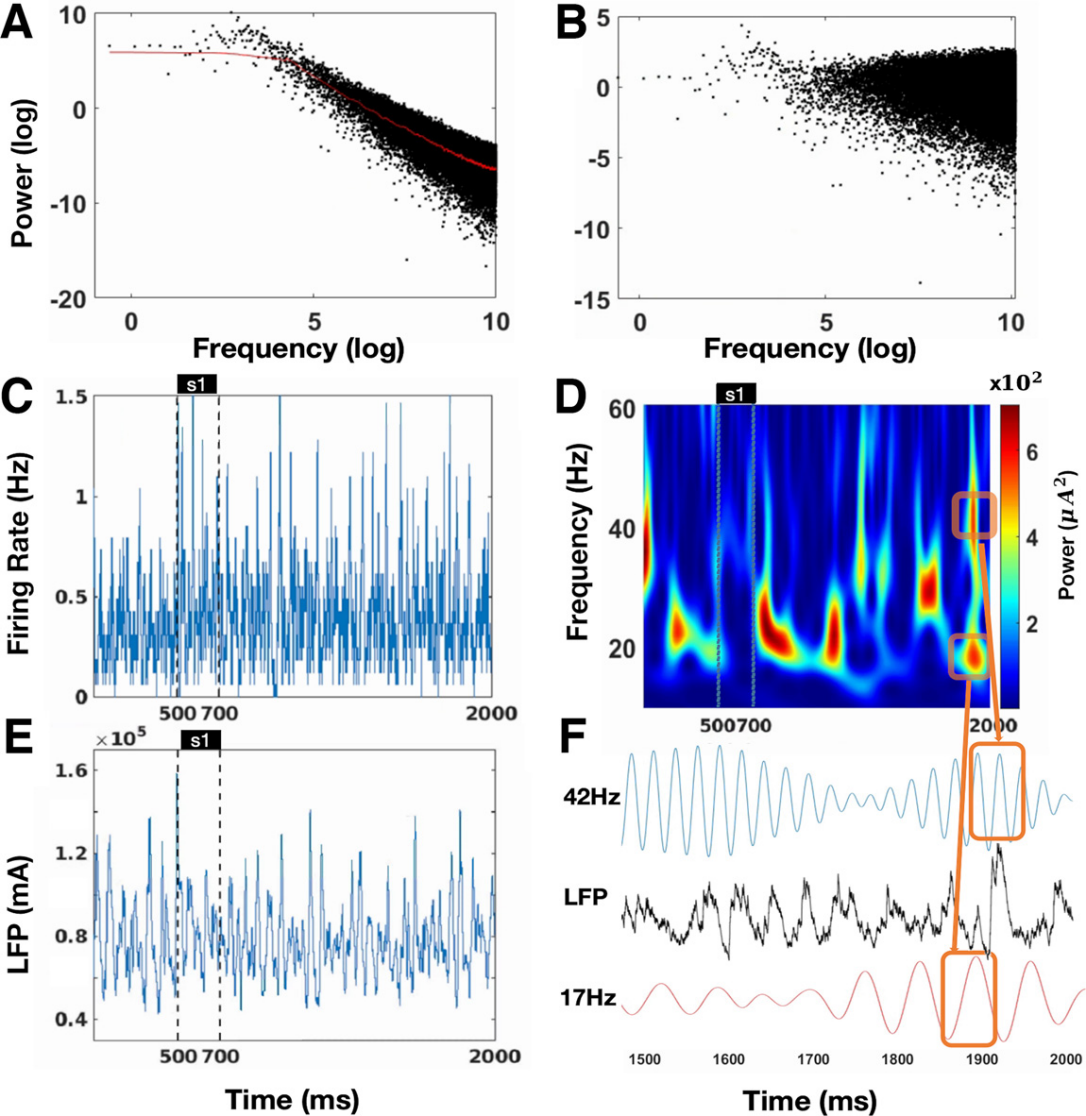
The oscillation in the network. ***A***, The power of raw simulated LFP of the network. The black dots plot the power against frequency on a logarithmic scale for LFP and has a power-law distribution. The red curve represents the 250-point average smooth curve of the log-scaled power and the log-scaled frequency. ***B***, The power of LFP normalized by the red curve in panel ***A***. ***C***, Example of the population firing rate of pyramidal cells in a single trial. ***D***, Example of the spectrogram of normalized LFP shown in panel ***C*** based on Morlet wavelet. ***E***, Approximated LFP in single trial as in panel ***C***. ***F***, Zoom-in on the approximated local field potential around the time of γ and β burst as seen in the spectrogram in panel ***D***. Blue curve shows the local field potential filtered at 42 Hz, and red curve shows local field potential filtered at 17 Hz.

We approximated the local field potential (LFP) using the total synaptic currents onto pyramidal cells following the method proposed by Mazzoni et al. (2015):

(24) $$\text{LFP}\left(\text{t}\right)=\underset{\text{pyr}}{\sum }[{\text{I}}_{\text{AMPA}}\left(\text{t}-{\text{τ}}_{\text{AMPA}}\right)+{\text{I}}_{\text{NMDA}}(\text{t})-1.65\times {\text{I}}_{\text{GABA}}\left(\text{t}\right)],$$

where ${\text{τ}}_{\text{AMPA}}=6$ms. We applied a one-dimension filter with a window size of 4 ms to the LFP and calculated the power spectrogram of LFP using continuous wavelet transform with Morlet wavelet. The background energy of our model demonstrates the 1/f characteristic for high frequency oscillation (refer to Miller and Wang, 2006; Wang, 2010). We first computed the power-frequency correlation of our model without external input shown in Figure 3*A*. We observed that the power for low-frequency oscillation remains almost constant, but decays in accordance with a power-law trend. The power-frequency data were smoothed using an average of 250 points and represented by the red line to signify the background power of oscillation. We subtracted the value of the red line from the simulated power. Figure 3*B* illustrates the power of LFP, which has been normalized by the red curve in Figure 3*A*. This normalization is applied in subsequent spectral analysis to obtain the normalized power.

#### Burst rate calculation

A burst in a particular frequency is defined as an interval when spectral power exceeds twice the standard deviation above the average value of that frequency and lasts for at least three cycles (Lundqvist et al., 2016). After we located the bursts in a single trail, we applied the same process to multiple trails and calculated the frequency of the bursts in the β (10–30 Hz) or γ (35–100 Hz) band over time. Similar to how we extracted the firing rate, we filtered the calculated burst frequency with a one-dimensional Gaussian filter, with a window size of 60 ms, to approximate the burst rate for a certain frequency range.

#### Statistical comparison

In our study, we employed independent sample *t* tests and ANOVA to compare between groups after conducting tests for homogeneity of variance and making necessary corrections. We also provided the effect size and confidence interval for each significant test result. When necessary, we conducted multiple comparison analyses to offer comprehensive results. All statistical analyses were performed using MATLAB. Our model generated substantial data from multiple simulations with high power and narrow confidence intervals. Based on the results from the online power and sample size calculator (https://www.gigacalculator.com/calculators/), the power for all statistical tests were close to 100%.

## Results

### The continuous spiking network can hold concurrent and sequential cues

**Figure 4.**
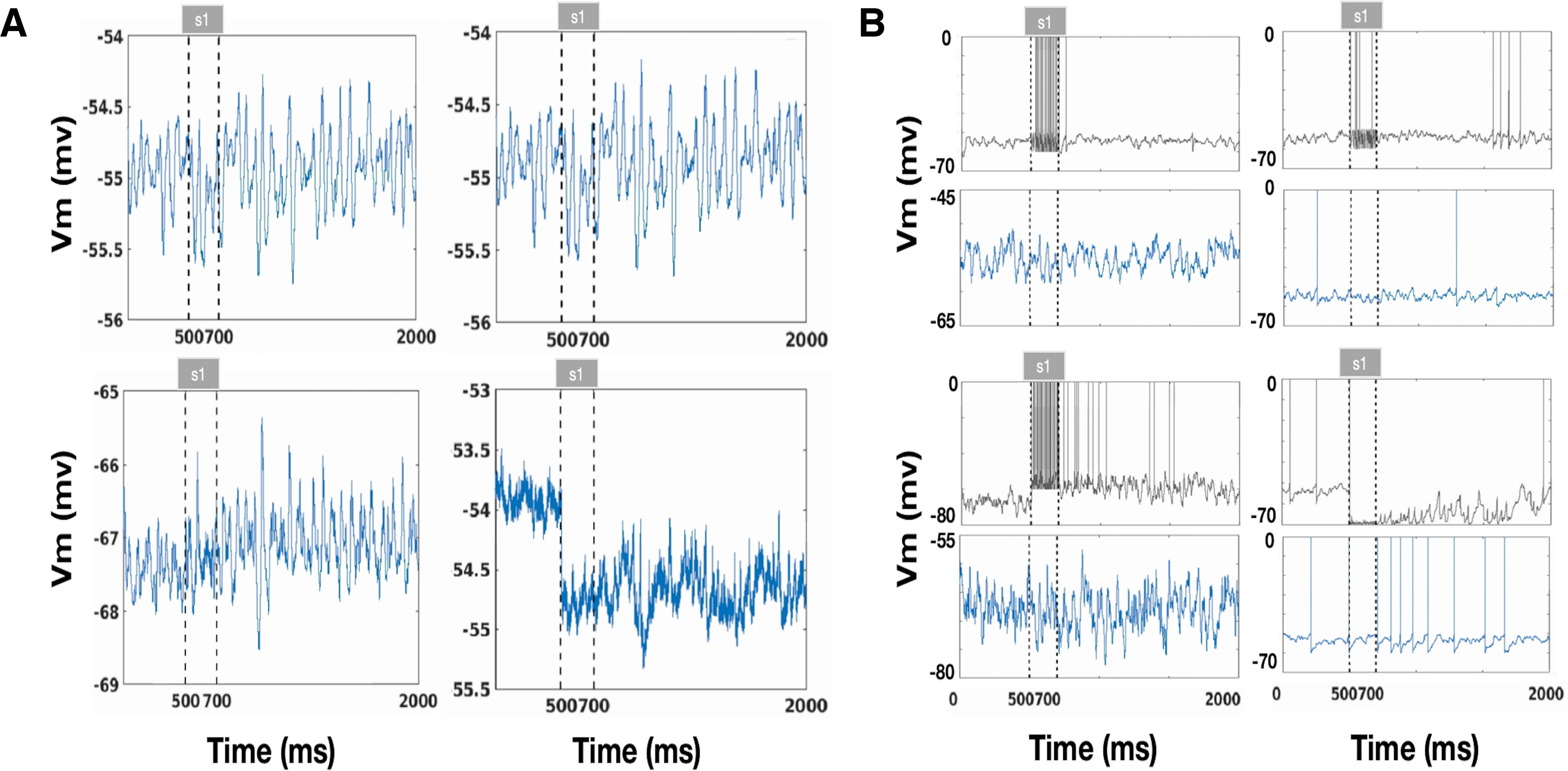
Voltage traces from neurons. ***A***, Average membrane potential for pyramidal dendrites (top left), somas (top right), FS cells (bottom left), and nFS cells (bottom right) in response to one cue. Dotted lines indicate the presence of the stimulus. ***B***, Example of single-cell membrane potential for pyramidal dendrites (top left), somas (top right), FS cells (bottom left), and nFS cells (bottom right) in the preferred and nonpreferred direction in response to a cue. The voltage trace of neurons in the preferred direction is represented by the black line and those in the nonpreferred direction by the blue line.

We considered a spiking neural network consisting of one population of 4098 pyramidal neurons, one population of 512 FS, and one population of 512 nFS. Pyramidal cells are uniformly distributed on a ring according to their preferred cue angles (Fig. 1*A*; Compte et al., 2000), mimicking the columnar organization of the monkey PFC (Goldman-Rakic, 1995; Rao et al., 1999; Constantinidis et al., 2001). Using this continuous spiking network model, we investigated how WM manipulates sequential and concurrent cues. We first presented one direction to the network for a brief period and then withdraw the cue to explore the spatiotemporal pattern of neural activity elicited by the cue presentation. As shown in Figure 2*A*, pyramidal cells showed relatively sparse and regular discharges in the absence of stimulus from 0 to 500 ms. At 500–700 ms, the stimulation elicited an intense firing in the cued direction. When the cue was withdrawn, this intense discharge in the preferred direction persisted and showed oscillatory activity throughout the 700- to 2000-ms delay period. We call this sustained oscillatory activity in the cued direction “oscillatory activity bump” and believe it maintains information about the cue. Pyramidal cells in other directions were unaffected during and after the cue presentation and continued to discharge sparsely and regularly. The inhibitory interneurons nFS (Fig. 2*B*, blue) and FS (Fig. 2*B*, red) showed opposite trends. In the prestimulus period (0–500 ms), the nFS discharged as sparsely and regularly as the pyramidal cell, whereas the FS barely discharged. Within 500–700 ms of stimulus presentation, the nFS stopped firing in the preferred direction, whereas the FS began to fire strongly and intensely in the preferred direction. This firing pattern continued throughout the 700- to 2000-ms delay period after stimulus withdrawal. The stimulus had little effect on the nonpreferred nFS and FS, and their spikes remained sparse or absent. Figure 4*B* provides additional information about the average membrane potential of the cells. It also shows examples of pyramidal cells, FS, and nFS in the preferred and nonpreferred directions. We then presented two or three directional cues to the network sequentially or concurrently. Given concurrent cues, the network simultaneously elicits two or three distinct oscillatory activity bumps that persist throughout the delay period (Fig. 2*C*,*D*), and each distinct activity bump maintains the information of one cued direction. For sequential cues, the network elicits distinct oscillatory activity bumps one after another (Fig. 2*E*,*F*). The later presented cue evokes a new persistent oscillatory activity bump and does not disrupt the persistent oscillatory activity bump elicited by the previous cue. These results show that our continuous network can store not only concurrent information but also sequential information of WM.

### Labor division of interneurons in γ and β oscillation

**Figure 5.**
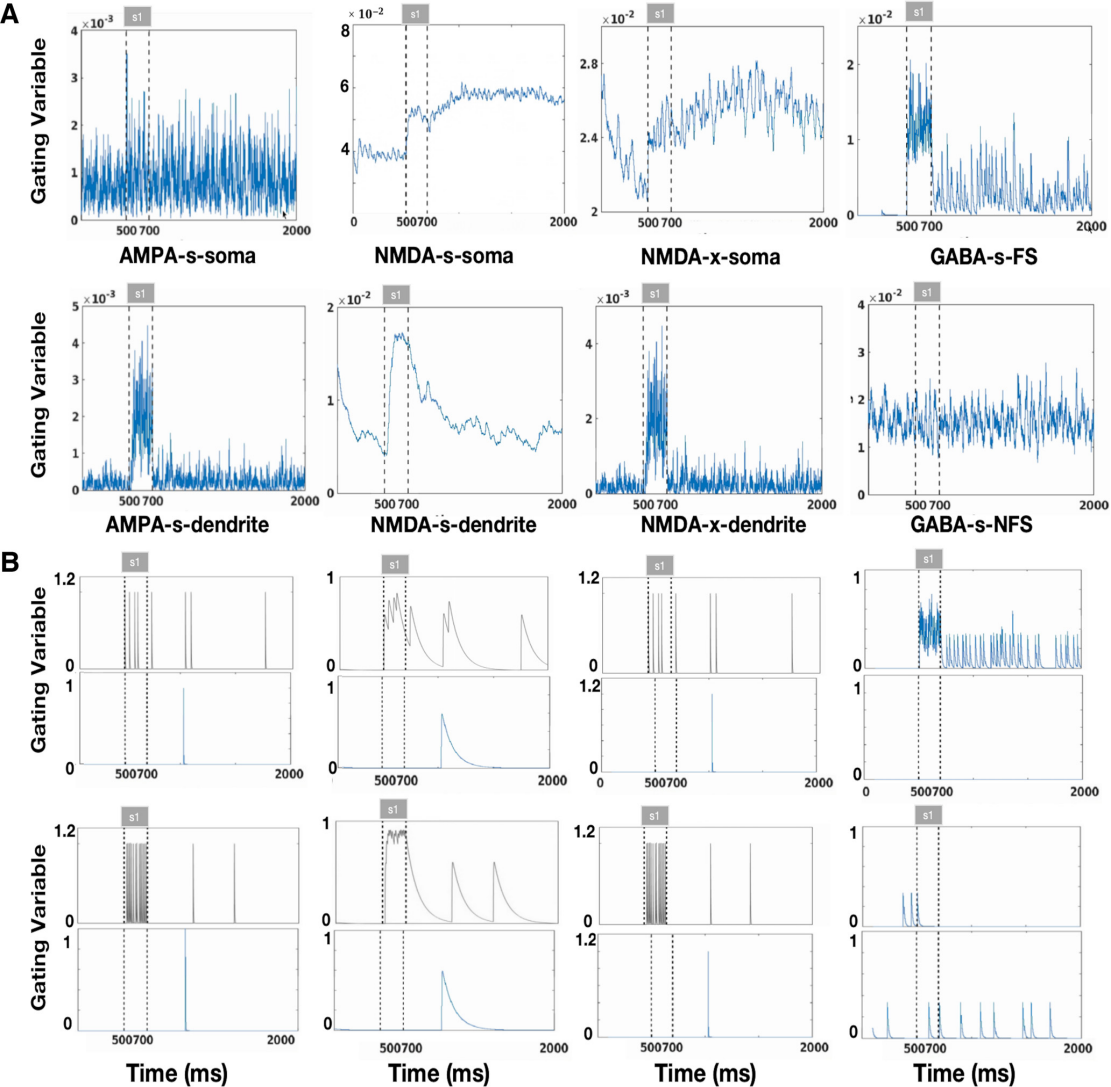
Gating variables form neurons. ***A***, Average AMPA, NMDA, and GABA gating variables associated with presynaptic pyramidal dendrites, somas, FS cells and nFS cells in response to one cue. Dashed lines indicate the presence of the stimulus. ***B***, Example of single cell gating variable in response to one cue. The gating variables of individual neurons are shown at the same positions as the corresponding average gating variables in ***A***. The gating variables of neurons in the preferred direction are represented by the black line and those in the nonpreferred direction by the blue line.

**Figure 6.**
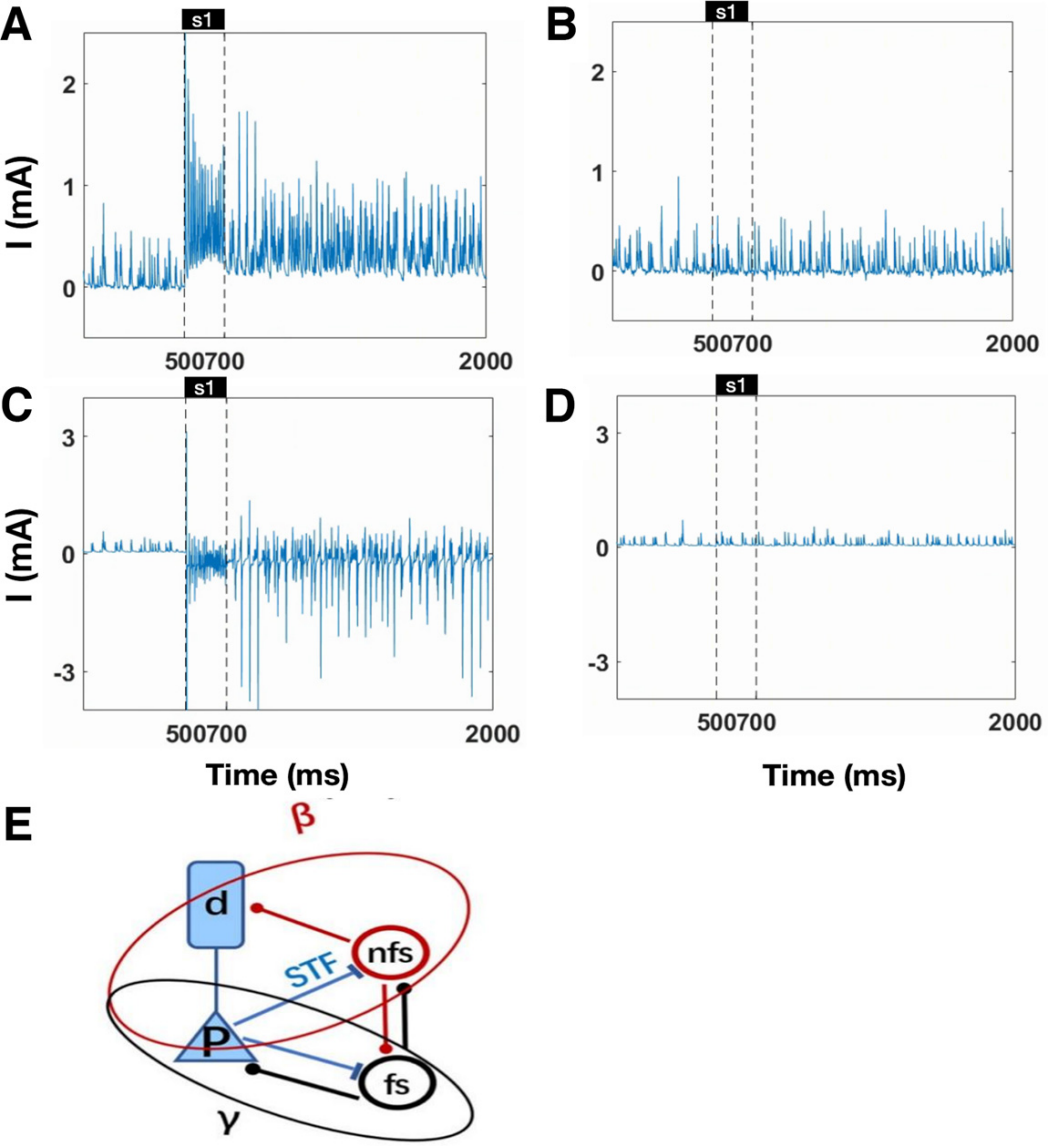
Labor division of FS and nFS interneuron. ***A***, The total synaptic input to FS close to the cued direction. ***B***, The total synaptic input to FS far away from the cued direction. ***C***, The total synaptic input to nFS close to the cued direction. ***D***, The total synaptic input to nFS far away from the cued direction. ***E***, The schematic diagram of labor division of interneurons in the γ band and β band oscillation. Interaction between pyramidal cell and nFS results in β band oscillation, while interaction between pyramidal cell and FS leads to γ oscillation.

To investigate the oscillatory behavior in WM, we calculated the firing rate of pyramidal cells and the LFP given one directional cue in a single trial (Fig. 3). The population firing rate and LFP oscillate before and after the cue presentation and throughout the delay period (Fig. 3*C*,*E*). The spectrogram of the LFP shows that the network oscillates in the β band before the cue presentation but oscillates in the β and γ bands during the delay period (Fig. 3*D*,*F*). Figures 4 and 5 provide an overview of the average membrane potential (Fig. 4*A*) and gating variables (Fig. 5*A*) during a single trial for different neurons or compartments. In addition, these figures provide examples of individual neurons in both preferred and nonpreferred directions (Figs. 4*B*, 5*B*). Overall, the mean membrane potential of pyramidal cells [Mpre = −55.054, Maft = −54.918, *t* = −43.399, *p* < 0.001, *d* = 0.430, cl = [−0.142,−0.130] (Fig. 4*A*), where Mpre is the average membrane potential before cue presentation and Maft represets that after the cue] and FS (Mpre = −67.212, Maft = −67.117, *t* = −24.981, *p* < 0.001, *d* = 0.224, cl = [−0.103,−0.088]; Fig. 4*A*) increased, and that of nFS cells decreased after stimulus presentation (Mpre = −53.999, Maft = −54.598, *t* = 542.997, *p* < 0.001, *d* = 3.464, cl = [0.596,0.601]; Fig. 4*A*). We calculated the total synaptic currents received by the interneurons (Fig. 6). We found that the cue presentation increases the synaptic input to FS close to the presented direction (Mpre = 0.057, Maft = 0.330, *t* = −271.328, *p* < 0.001, *d* = 1.360, cl = [−0.274,−0.270]; Fig. 6*A*) and activates these FS (Fig. 2*B*) during the cue presentation and throughout the delay period. Cue presentation has little effect on the synaptic input to FS far away from the cued direction (Mpre = 0.049, Maft = 0.041, *t* = 13.920, *p* < 0.001, *d* = 0.092, cl = [0.007,0.009]; Fig. 6*B*) and cannot activate them (Fig. 2*B*). We found that synaptic currents input to nFS near the cued direction decrease because of the inhibition from active FS during cue presentation and the delay period (Mpre = 0.081, Maft = −0.215, *t* = 188.834, *p* < 0.001, *d* = 0.820, cl = [0.293,0.299]; Fig. 6*C*), and these nFS are suppressed (Fig. 2*B*). However, the input to nFS far away from the cued direction does not change much (Mpre = 0.079, Maft = 0.080, *t* = −3.175, *p* = 0.002, *d* = 0.021, cl = [−0.002,−0.000]; Fig. 6*D*). In summary, before the cue presentation, weak excitation is insufficient to activate FS, and the interaction between pyramidal cells and nFS dominates the network activity, resulting in β band oscillation. The cue presentation induces strong excitation and activates FS, and the interaction between pyramidal cells and FS leads to γ band oscillation in the network. Therefore, these results show a division of labor between FS and nFS in γ band and the β band oscillations, as shown in Figure 6*E*.

### γ Power increases in the cued direction

**Figure 7.**
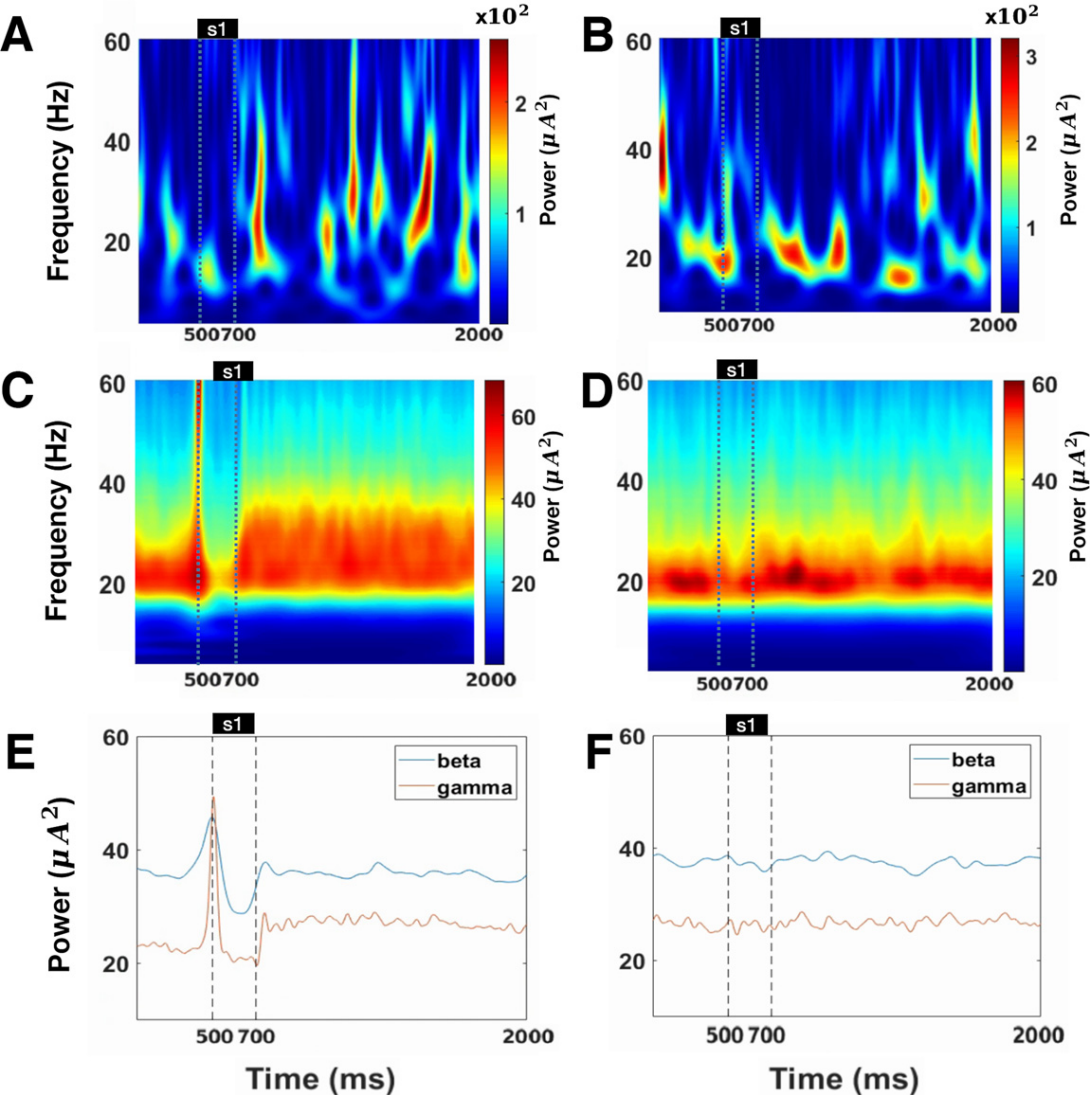
Oscillation power in the preferred direction and anti-preferred direction. ***A***, The spectrogram for LFP approximated from the pyramidal cells preferring the cued direction in a single trial. ***B***, The spectrogram for LFP approximated from the pyramidal cells far away from the cued direction in a single trial. ***C***, The average of spectrogram for preferred direction over 900 trials. ***D***, The average of the spectrogram for anti-preferred direction over 900 trials. ***E***, The average normalized power (β band:10–30 Hz, γ band: 35–100 Hz) for the preferred direction. The red line denotes the γ power, and the blue line denotes the β power. ***F***, The average normalized power for nonpreferred direction. The red line denotes the γ power, and the blue line denotes the β power.

Experiments (Pesaran et al., 2002) showed that the increase in γ band power was sustained during the delay period in the preferred direction, while γ band power did not change in the anti-preferred direction. We calculated the LFP based on the pyramidal cells close to the cued direction, which is the preferred direction, and the pyramidal cells far away from the cued direction, which is the anti-preferred direction. We found that in the preferred direction, the LFP mainly consists of β band oscillation before the cue presentation, and a strong γ band oscillation appears after the cue presentation in a single trial (Fig. 7*A*) or averaged over trials (Fig. 7*C*). The power of γ band increases significantly (Mpre = 23.942, Maft = 26.616, *t* = −80.835, *p* < 0.001, *d* = 0.784, cl = [−2.739,−2.609]), but the power of β band decreases (Mpre = 37.157, Maft = 35.381, *t* = 76.696, *p* < 0.001, *d* = 0.737, cl = [1.731,1.822]; Fig. 7*E*). There is little increase in γ band oscillation (Mpre = 26.370, Maft = 26.741, *t* = −54.777, *p* < 0.001, *d* = 0.479, cl = [−0.385,−0.358]; Fig. 7*B*) for the anti-preferred direction, and the β band oscillation is not decreased (Mpre = 37.752, Maft = 37.450, *t* = 47.799, *p* < 0.001, *d* = 0.348, cl = [0.289,0.314]; Fig. 7*F*). Note that there was an early increase in γ and β band power in Figure 7*E*,*F*. We think this was an artifact of the cwtft algorithm. CWTFT calculates the power of a signal by locally integrating the multiplication of signals with shiftable symmetric wavelets of different window sizes and center frequencies. In the same wavelet window, wavelet analysis is isotropic, thus unable to distinguish components before and after stimulus presentation. When the signal frequency undergoes significant changes within a certain window, the window that contains the changing signal cannot differentiate between the prechange and postchange components, showing an earlier increase in power (similar to Kaiju et al., 2017). In short, cue presentation increases the power of the γ oscillation and decreases β oscillation in the preferred direction (Fig. 7*E*,*F*), which are consistent with the experimental observations (Pesaran et al., 2002).

### WM load enhances the γ power during the delay period

**Figure 8.**
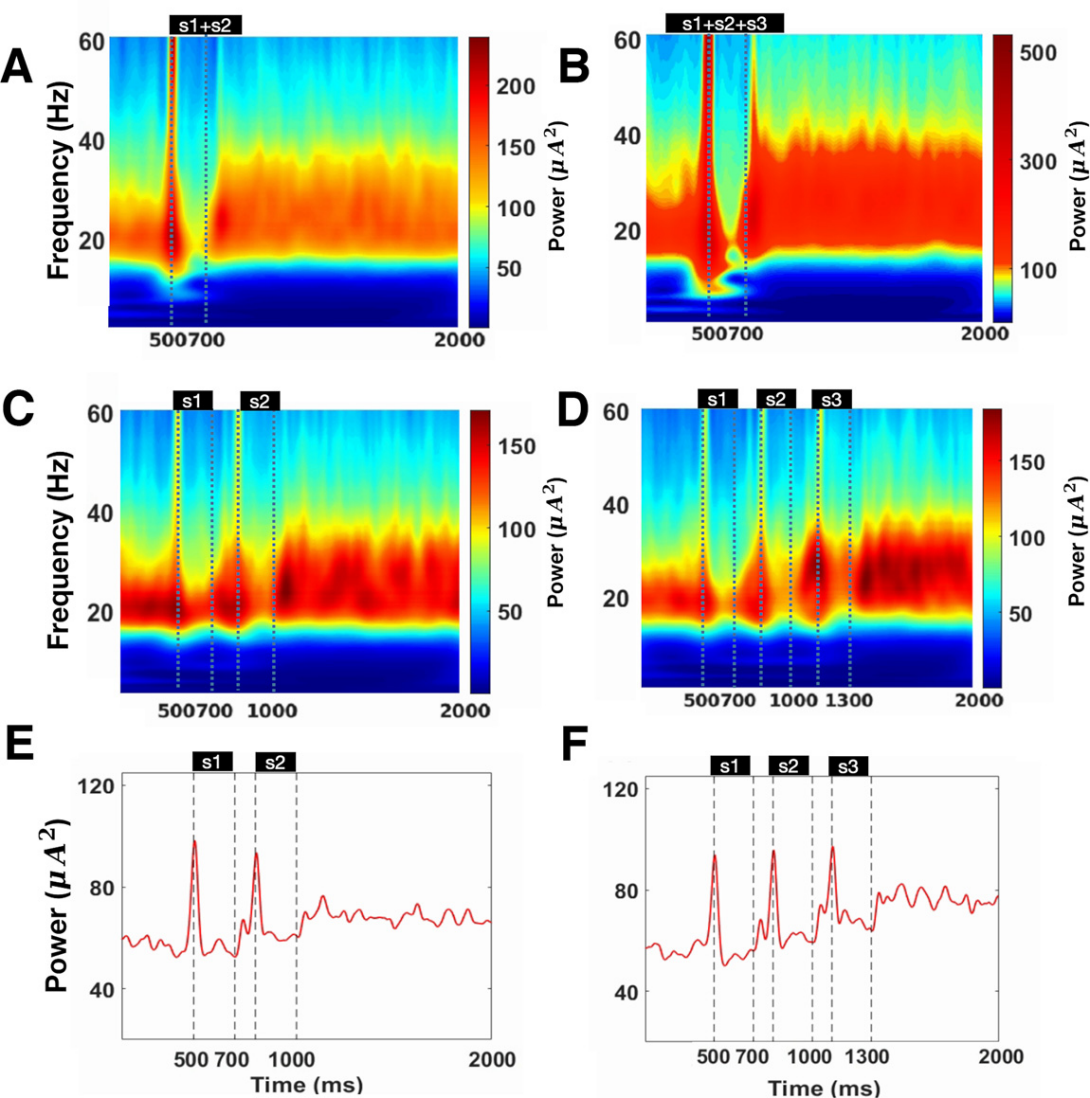
The average spectrogram of LFP given concurrent or sequential cues over 500 trials (***A–D***) and the average normalized γ power for sequential cue conditions (***E***, ***F***). ***A***, The average spectrogram for two concurrent cues. ***B***, The average spectrogram for three concurrent cues. ***C***, The average spectrogram for two sequential cues. ***D***, The average spectrogram for three sequential cues. ***E***, The average normalized γ power for two sequential cues. ***F***, The average normalized γ power for three sequential cues.

We further analyzed the spectrogram of LFP given concurrent or sequential cues. The average spectrogram of LFP over 500 trials exhibits several characteristics. First, as previously shown, the network exhibits β band oscillation before the cue presentation, and the β power tend to decreases after cue presentation (two concurrent cues: Mpre = 107.469, Maft = 102.083, *t* = 39.865, *p* < 0.001, *d* = 0.412, cl = [5.121,5.651]; three concurrent cues: Mpre = 117.394, Maft = 108.605, *t* = 25.929, *p* < 0.001, *d* = 0.272, cl = [8.124,9.453]; two sequential cues: Mpre = 100.995, Maft = 97.639, *t* = 83.571, *p* < 0.001, *d* = 0.549, cl = [3.277,3.435]; three sequential cues: Mpre = 98.238, Maft = 98.990, *t* = −15.451, *p* < 0.001, *d* = 0.085, cl = [−0.847,−0.657]). Second, the network elicits higher γ band oscillation (around 60–70 Hz) on cue presentation (two concurrent cues: Mpre = 62.763, Maft = 68.745, *t* = −43.986, *p* < 0.001, *d* = 0.450, cl = [−6.249,−5.715]; three concurrent cues: Mpre = 68.370, Maft = 75.840, *t* = −21.950, *p* < 0.001, *d* = 0.233, cl = [−8.137,−6.803]; two sequential cues: Mpre = 58.800, Maft = 66.297, *t* = −133.050, *p* < 0.001, *d* = 1.081, cl = [−7.607,−7.386]; three sequential cues: Mpre = 58.202, Maft = 70.287, *t* = −193.409, *p* < 0.001, *d* = 1.299, cl = [−12.207,−11.963]) and γ band oscillation maintains during the delay period. Third, the γ power remains almost constant during the delay period for concurrent cues (Fig. 8*A*,*B*). In contrast, the γ power increases along with the arrival of new cues in sequential situations and the power is maintained throughout the delay period (two sequential cues: *F* = 13,057.29, *p* < 0.001; ${\text{η}}^{2}$= 0.225, three sequential cues: *F* = 24,175.79, *p* < 0.001, ${\text{η}}^{2}$= 0.446, with multiple comparisons *p* < 0.001; Fig. 8*C*,*D*). We show the average γ power in sequential trials in Figure 8*E*,*F*. The trajectory of γ power is similar to the results shown by Lundqvist et al. (2016; their Figure 6), albeit with a slight distinction: the γ band exhibited an increase following the presentation of the second stimulus before the presentation of the third stimulus.

**Figure 9.**
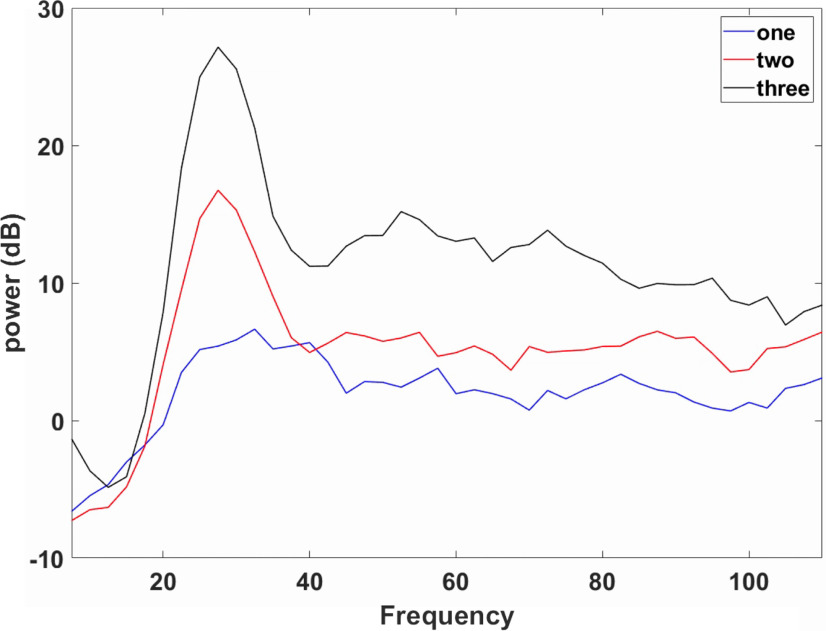
The normalized power of local field potential. γ power induced by two concurrent cues (red line) is higher than that induced by one cue (blue line) but lower than that resulting from three concurrent cues (black line).

We performed a Fourier analysis on the LFP given concurrent cues, and the results are consistent with the spectrogram analysis. γ power increases with the WM load (*F* = 71.06, *p* < 0.001; Fig. 9). Three cues lead to the highest γ power and one cue results in the lowest γ power. The mean of the γ power are M1cue = 0.493, M2cue = 1.060, and M3cue = 2.357 for 1, 2, and 3 cues, respectively (multiple comparisons *p*12 = 0.002, *p*13 < 0.001, *p*23 < 0.001; Fig. 9). The dependence of γ power on WM load can be explained by network activity. Strong excitation induced by cue presentation activates high-threshold FS and the interaction between pyramidal cells and FS results in γ oscillation. One cue elicits one distinct activity bump with γ oscillation and more cues elicit more distinct activity bumps with γ oscillation (Fig. 2), suggesting that more neurons participate in γ oscillation and result in higher γ power.

### Brief bursts of narrow-band γ and β oscillation in a single trial

**Figure 10.**
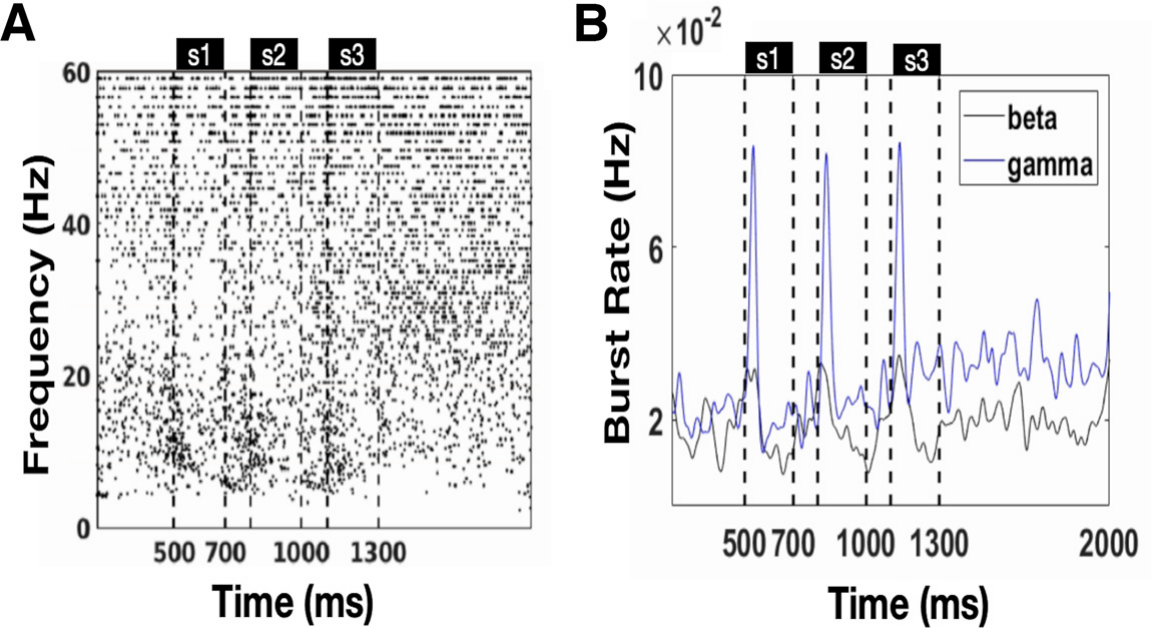
The oscillatory bursts. ***A***, The raster plot shows the occurrence of oscillatory bursts in 500 trials of simulation. ***B***, The rate of β and γ bursts.

Although the raster plots of spiking in Figure 2 show oscillatory activity, the single spectrogram of LFP (Figs. 3*B*, 7*A*,*B*) shows a different scenario: γ and β oscillations occurred irregularly in the form of short bursts that are narrow and variable in frequency. This observation is similar to a previous monkey experiment (Lundqvist et al., 2016). Here, we identified the γ and β bursts using the algorithm proposed in the reference (Lundqvist et al., 2016). We identified each burst in 500 trials with sequential cues and plotted each burst as a point in Figure 10*A*. We also calculated the β and γ burst rates (Fig. 10*B*). The β burst rate decreases slightly after cue presentation (Mpre = 0.0207, Maft = 0.0196, *t* = 21.08, *p* < 0.001; Fig. 7*F*) and remains almost constant during the delay period (Fig. 10*B*). The γ burst rate increases when the cue is presented (Mpre = 0.0570, Maft = 0.0685, *t* = −48.93, *p* < 0.001; Fig. 7*F*) and increases with increasing WM load (*F* = 229.69, *p* < 0.001, multiple comparisons *p* < 0.001; Fig. 10*B*). During the delay period, the γ burst shows a slightly increasing trend, which is consistent with the experimental observation by Lundqvist et al. (2016; their Figure 8B).

## Discussion

We have developed a working memory model that utilizes a continuous attractor structure based on the bistable excitatory-inhibitory microcircuit consisting of FS, nFS, and pyramidal cells in PFC. The main contributions of our work are threefold. First, the proposed model can maintain the information of sequentially presented cues and concurrently presented cues. Previous oscillatory and dynamic models collapse in the presence of concurrent information because these models rely on the oscillatory phase or timing to encode the information (Constantinidis et al., 2018). The persistent activity model has not been used to manipulate sequential information (Edin et al., 2009; Wei et al., 2012). Second, we identified the mechanism underlying the enhancement of γ power in the WM task. Cue presentation elicits strong excitatory recurrent synaptic currents that activate high-threshold FS. The interaction between pyramidal cells and FS leads to γ oscillations and enhances γ power during the delay period. Third, we demonstrated the division of labor between high-threshold FS and nFS for oscillation in the WM. The interaction between nFS and pyramidal cells leads to β oscillation, while the interaction between FS and pyramidal cells leads to γ oscillation.

We showed that the transient and sparkling γ and β burst in a single spectrogram of LFP can be approximated from the persistent oscillatory activity of the network (Fig. 3*B*), and the γ burst rate increases with the WM load (Fig. 8*B*). The discrete and sparkling γ and β burst in a single LFP spectrogram has been considered as evidence that WM is manifested through transient or discrete oscillatory dynamics, rather than sustained firing (Lundqvist et al., 2016). However, our results indicate that transient and sparkling γ and β bursts in a single LFP spectrogram may indeed result from persistent oscillatory firing. Therefore, our results favor the theory of persistent activity underlying WM.

In this work, we have demonstrated the division of labor between FS and nFS in generating γ and β oscillation. The interplay between high-threshold FS and pyramidal cells results in the γ oscillation, whereas the interaction between nFS and pyramidal cells leads to the β oscillation. Before the cue presentation in WM tasks, the weak recurrent excitation from spontaneous discharges of pyramidal cells can activate low-threshold nFS but not the high-threshold FS, resulting in β oscillation. Cue presentation evokes strong activity of pyramidal cells and strong recurrent excitation to activate FS. As a result, the network exhibits γ oscillation. This observation is consistent with experimental results that optogenetic activation of FS induces γ rhythm (Cardin et al.,2009) and the spiking of somatostatin (SOM) and parvalbumin (PV) cells differentially correlates with β and γ oscillations and activation of PV cells enhances γ oscillation in V1 area (Chen et al., 2017).

We computationally identified that γ power can be enhanced by recruiting more high-threshold FS because of the stronger excitation evoked by the cue presentation in WM. This mechanism may be common to other cognitive processes, such as attention and sensory information processing in perceptual decision-making. It has been extensively observed in cognitive processes such as WM (Jensen et al., 2007; Yamamoto et al., 2014), attention (Engel et al., 2001; Fries et al., 2001; Jensen et al., 2007; Fries, 2009; Gregoriou et al., 2009; Kim et al., 2016), and sensory information processing in perceptual decision-making (Frien et al., 2000; Siegel and König, 2003; Kayser and König, 2004; Henrie and Shapley, 2005; Liu and Newsome, 2006; Berens et al., 2008). Studies had observed that the selected neuronal population increased their firing rate when attention was directed to their receptive field (Luck et al., 1997; McAdams and Maunsell, 1999; Bichot et al., 2015; Thiele et al., 2016), implying strong excitation and potentially recruiting more FS neurons. γ oscillations in the sensory cortex are often considered as a proxy for the encoding of sensory evidence during perceptual decision-making because of visual γ band activity and its dependence on stimulus strength and various stimulus features (Frien et al., 2000; Siegel and König, 2003; Kayser and König, 2004; Henrie and Shapley, 2005; Liu and Newsome, 2006; Berens et al., 2008). Based on these, we hypothesize that sensory input would evoke a stronger excitation in the sensory cortex, which could recruit more FS and increase the γ power.

Worth noting that the synapses from pyramidal cells to the nFS are dynamic synapses with short-term potentiation (STP). STP plays an essential role in the modulation of γ and β bursts. In the biological brain, there is short-term synaptic facilitation between pyramidal cells and nFS and short-term synaptic depression between pyramidal cells and FS. These short-term plasticities allow the brain to respond differently to afferents of different durations. Brief currents cause γ oscillations, while prolonged currents cause β oscillations. Based on this mechanism, these observations are reproduced in previously published work (Feng et al., 2021). In our simulation, we only briefly present the stimulus to the model, resulting in oscillations at the γ frequency, and short-term synaptic facilitation between pyramidal cells and nFS helps control excitability in our model. The synapses equipped with STP can temporarily increase inhibition by increasing the effective excitation from the pyramidal cells to the nFS and expanding the range of parameters to keep excitation and inhibition in balance. Too much excitation can lead to the merging of different information (Wei et al., 2012) or false memories (Edin et al., 2009), while too much inhibition can prevent the network from maintaining information. Suppose the synapses from pyramidal cells to the nFS are fixed without STP; the network is prone to produce spurious bursts of activity because of too much excitation or to forget the information because of too much inhibition. At present, we have not considered the effects of prolonged stimulus. It would be interesting to investigate how the brain adapts and responds to prolonged stimulus in the future.

## Synthesis

Reviewing Editor: Niraj Desai, National Institute of Neurological Disorders and Stroke Decisions are customarily a result of the Reviewing Editor and the peer reviewers coming together and discussing their recommendations until a consensus is reached. When revisions are invited, a fact-based synthesis statement explaining their decision and outlining what is needed to prepare a revision will be listed below. The following reviewer(s) agreed to reveal their identity: NONE.

Two reviewers and I read the revised manuscript and the rebuttal letter. While we all agree that the manuscript has considerable merit and would be valuable contribution to the literature, we also think it still needs major revision. One of the reviewers wrote a detailed list of concerns (appended below) that I would urge you to consider and address carefully should you choose to resubmit.

REVIEWER COMMENTS

This study successfully reproduces Gamma and Beta oscillations in a working memory test with concurrent cues by a spiking model with pyramidal cells and two types of interneurons. Importantly, the work contributes to the debate on whether a sustained activity or short-term plasticity is a more essential mechanism of working memory by showing that variable rhythmic activity can also be achieved through a model with bump attractors. The work is novel, and I believe it can make an impact in the field. However, this manuscript still requires detailed work to make conclusions convincing. I feel like reading a promising working progress report but not a combined polished manuscript. I have to guess what the authors mean in several places. A major revision is needed before publication.

One major concern is about the spectrum analyses. The results and the related arguments are intuitively right, but so far unconvincing. Compared to the previous version, the author claimed to reproduce an increase in the gamma range during the stimuli presentation. However, these results only appear in the rebuttal to the reviews rather than in the major figures of the main manuscript. In fact, even that figure, on the bottom left and right, the Gamma power should be comparable before the onset of the third stimulus at around time=1000ms. However, the gamma power almost doubles on the right compared to the left. Why that is the case? As Lundqvist showed in the top left and right panels, the gamma power should be consistent before S3 presents. This may be due to different random seeds. In this scenario, several runs should be combined into the analysis, but not let the readers wonder whether a cherry-picked result is shown.

Furthermore, with a closer look, the gamma power increases before the stimuli onset. This is not observed in the experiment and is not reasonable from a modeling aspect, since the statistic of the gamma rhythm should be the same before the stimuli onset. I suspect the results may come from a relatively large sliding window when generating the spectrograms and a relatively small time constant of the network dynamics. On the contrary, Figures 2, 4, 5, and 10 consistently showed that all the observable changes happen after the stimuli onset. If this is indeed an artifact, please fix it. If not, please explain the temporal differences between different analysis protocols convincingly.

Last, an accurate spectrum analysis requires subtracting 1/f^a background energy. For one example, see the discussion at Wang 2010, Physiol. Rev. Fig. 15B. I suspect the conclusion drawn from Figure 7 C to F and Figure 9 would be different if the authors did it correctly.

Another major concern is the quality of the manuscript. Little issues constantly pop up to bother me when I read the manuscript. Some examples are here: In the methods section, the parameters have three digits but without references or explanation. The authors should include the references from the Rebuttal. Even better, but not required, authors should include some recent references. Some of them argued the lateral innervation width of interneurons may be comparable with that of excitatory neurons. Further, the results in all the major figures are described with realistic units, but the parameters have no units associated when they are described in the Method section. Please include the units for all the parameters in future versions. In the figures, the direction of the y-axis labels is not consistent, and the figure quality is low. A thorough round of editing, maybe with the suggestions generated from a Large Linguistic Model, would help in the future submission.

A comparable minor thing, the authors misunderstood the meaning of a convergence test. A convergence test is generated by fitting the error line to the timestep in the log-log scale. To be specific, the author should check whether the error decay to one-fourth or one-half if decrease timestep by half. See https://math.stackexchange.com/questions/3115199/measuring-the-convergence-order-of-a-numerical-scheme-for-pde. If authors succeed in generating a 2-nd order scheme, the slope of the fitting should be 2.

Some minor things I found:

Line 65, “Fs” should be FS

Line 101 to 106, personal suggestion: “Without losing generality, a network of the size of 2^n can be utilized by the FFT”

Line 111 delete one “equation” from two “equation”. The font size is not consistent, either.

Line 134, for better visualization, the parameters should be included in a table.

Line 146, is the dendrite voltage reset to -60 as well when the neuron spikes? In realistic neurons, the reset is not that strong or even negligible. See papers from Matthew Larkum.

Line 149, is p1=p2? It should be.

Line 221, equation 20 is not mathematically correct. Rewrite 1[rand > dt ∗ rate] in a standard Poisson process with \lambda=1

Line 289, Why is it unfair? Can you modify the analysis code such that comparing “indirectly” but “fair”?

Line 341, figure 5, is not sufficiently explained. This may raise questions from readers. For example, why average gating variable NMDA_s_soma is constantly elaborated, as in A top second, but not in either preferred direction or non-preferred direction, as in B top second?

Line 423, the authors may want first summarize the model.

Line 433, I am confused about the “division of labor.” What is “labor”? I don’t see any “labor” for nFS in this model. The nFS input does not show any change before and after the stimulus onset, while the beta power and burst rate change. Actually, will model results change if replacing nFS cells with a constant inhibitory input?

Line 475 Replacing “It is not difficult to imagine that” with “Based on these, we hypothesize that”

Line 485 Should be “These observations are reproduced in Fend et al. 2021”.

Line 536 after “GABA,” maybe add “averaging.” Is that right?

Line 537, before the “FS” cell, add “from”? “NFS” should be “nFS.”

## Author Response

Dear Editor,

Thanks for your comments and your patience. We also thank the reviewers for their constructive and helpful comments. Following the suggestions and comments of reviewers, we re-simulate the model using new parameters and the results are consistent with those shown in the original version of our manuscript. Attached please find the point-to-point response to reviewers’ comments.

Best

DH

Response to reviewers’ comments:

REVIEWER 1

The study succeeded in reproducing Gamma and Beta oscillations in a working memory test with concurrent cues by a spiking model with pyramidal cells and two types of interneurons. However, I am concerned about several issues not well addressed/described in the current draft.

1st, the connectivity between different pyramidal cells is way too narrow. In the original Compte et al.2000 that the author adapted, the connectivity Gaussian kernel has a width sigma_EE= 18 degrees, but in the current setup, it is just sigma_SS= 1 degree. The original parameter is chosen based on the tuning curve from experimental data but not chosen arbitrarily. The modification in the current model significantly reduces the interaction between the pyramidal cells that prefer different orientations. Essentially, the concurrent cues are stored by independent E populations in this model, which is not biologically plausible. In addition, the model losses the ability to explain other cognitive functions, such as decision-making.

Reply: Thank you for your advice. This parameter was chosen as 18 degree in original. Compte et. al.2000. In the revised version, we set parameter to 12.76 degree which is smaller than the original Compte et al.’s model, but is large enough for the mutliptle items working memory and is the same as in a multiple item working memory model by Wei et al. (2012) “From Distributed Resources to Limited Slots in Multiple-Item Working Memory: A Spiking Network Model with Normalization”. The results of our model have insignificant change and shown in the revised version of the manuscript. The new parameters are biologically more plausible than old version of our manuscript.

2nd, though the rhythmic behaviors in a WM task have not been well studied, several studies on the PV/SST and slow/fast oscillations have been established recently. However, these studies are not referred to or discussed in the manuscript. Some examples include, but are not limited to: Chen et al. 2017 (listed in the references but not referred to in the main context); Veit. J, .. Adesnik. H, 2017, Nature Neuroscience; Domhof. J, and Tiesinga. P, 2021, Neural computation; Hahn. G, .., Deco. G, 2022, eLife.

Reply: Thank you for the recommendation. We have incorporated these literatures in our revised manuscript, particularly, please refer to paragraph in INTRODUCTION section.

3rd, the authors claim that they reproduced the observed gamma rhythms during a WM task. However, in Lundqvist et al. 2016, fig. 6, the gamma power is strongest during the stimuli presentation but not during the delay period. This crucial mismatch is not explained in the paper.

Reply: thanks for the comments. We using the revised parameters and simulated the tasks. The new results (the bottom panels in the follow figure) we obtained are very similar to those reported by Lundqvist et al. 2016, with the highest gamma power during stimulus presentation. The gamma power during delay period increases with the items.

4th, the authors use the synaptic input to calculate the spectrum of LFP, but no voltage traces of pyramidal cells are shown. Surprisingly, even though only a small population of pyramidal cells (about 3 degrees) are storing the cue, their impact on the LFP is significant. I suspect this is because the inhibition current is so strong that it is no longer in a physical region. To illustrate this, plotting sample traces of voltage and gating variables is helpful.

Reply: We have shown the average membrane potential of neurons and that of example neuron and gating variables in Figures 4 and 5. Figure. 4 shows the average membrane potential for pyramidal dendrites, somas, FS cells, and NFS cells (Fig 4.A), with examples of neurons in preferred and non-preferred directions (Fig 4.B). Figure 5 shows mean gating variables over time for AMPA, NMDA, and GABA synapses (Fig 5.A), with examples of neurons in preferred and non-preferred directions (Fig 5.B).

Figure 4. Voltage traces from neurons. A) Average membrane potential for pyramidal dendrites (top left), somas (top right), FS cells (bottom left) and NFS cells (bottom right) in response to one cue. Dotted lines indicate the presence of stimulus. B) Example of single-cell membrane potential for pyramidal dendrites (top left), somas (top right), FS cells (bottom left) and NFS cells (bottom right) in the preferred and non-preferred direction in response to a cue. The voltage trace of neurons in the preferred direction is represented by the black line, and those in the non-preferred direction by the blue line.

Figure 5. Gating variables form neurons. A) AMPA, NMDA, and GABA gating variables for pyramidal dendrites, somas, FS cells and NFS cells in response to one cue. Dash lines indicate the presence of stimulation. B) Example of single cell gating variable in response to one cue. The gating variables of the individual neurons are shown at the same positions as the corresponding average gating variables in A). The gating variables of neurons in the preferred direction are represented by the black line, and those in the non-preferred direction by the blue line.

While neuronal and synaptic activity in the preferred direction is very pronounced, all neuronal activity and gating variables are within reasonable ranges.

5th, the model is not well explained in the method section. Many parameter values are missing in the whole manuscript. Further, how the noise is implemented is not described in the manuscript.

Reply:

Thanks for your advice! The Methods section has been updated, the model has been explained in detail, parameter descriptions have been added to the formula and code, and the noise implementation method has been explained.

6th, Since I have a question about how the model is simulated, I checked the code. The authors may disagree with me, but I don’t think the RK2 scheme is correctly implemented. At line 1130 of the WM_Oscil_git.m, the C_coeff and D_vec within the function RK2_4uNMDA are not evaluated at the half timestep but at the beginning of the timestep. This will introduce O(t) error to the scheme. Further, I noticed that the authors confused the value at the current step and the updated timestep. A few examples are on lines 600, 694, and 750. These mistakes can be avoided by storing these variables into a time-dependent vector or using the label _new, _old systematically in the code. Within those lines (694, 750), the scheme is forward Euler but not RK2, which makes the whole scheme 1st order but not 2nd order. I cannot debug the whole code, and I understand that some modification is reasonable in certain cases and can improve efficiency. If the authors believe what they did is correct, please do a convergent test and show the results. Or, the whole simulation should use the Euler scheme instead.

Reply:

Thank you for your advice, we have checked the code thoroughly.

1) In the function RK2_4uNMDA, C_coeff is equal to 1/syn_tau_f_NMDA, while D_vec is equal to 0. They are constant and take the same constant value at the beginning of the time step and at the half time step. The slopes (K1 and K2) at the half time step are C_coeff*(U_init-y_vec_temp) and U.*D_vec.*(1-y_vec_temp). They are updated when y_vec is updated to y_vec_temp.

2) On line 600, pyr_s_s_NMDA is incorrectly calculated with updated pyr_s_x_NMDA, we have corrected the error by saving the updated pyr_s_x_NMDA to pyr_s_x_NMDA_new.

3) On lines 694 and 750 pyr_s_Vm_new (pyr_d1_Vm_new) is correctly calculated with the current pyr_s_I_total. This is because pyr_s_I_total is calculated with pyr_s_I_leak, pyr_s_I_NMDA, and pyr_s_I_GABA, etc.,and they are all updated in the current step with pyr_s_Vm_prev from the last timestep.

We used the classical Foward-Euler LIF model to calculate the membrane potential and the second-order Range-Kutta method to integrate the gating variables. This choice is because changes in gating variables during neuronal firing are more complex and usually non-linear, whereas changes in the subthreshold membrane potential are approximately linear. We conducted a convergence test, providing 2 mV constant stimulation for 2000 ms. We found that for sufficiently small dt, the results obtained by the two methods are almost identical in 2000 ms of membrane potential computation. See the figures below.

Figure. Convergence test: The neurons were consistently stimulated with a 2mV stimulus for 2000 ms. A) The membrane potential of the neurons integrated with Euler (dt=0.02) and RK (dt=0.02) was almost identical, even in the last 40ms of the 2000ms stimulation (t=0.353, p=0.724). B) As the integration timestep is reduced, the error of the Euler method, measured as the difference in spike time, decreases. C) Similarly, as the integration timestep is reduced, the Euler method’s error, measured as the absolute difference in membrane potential, decreases.

Therefore, when the integration step equals 0.02, as in the experiment, there is almost no difference in accuracy between Euler and RK2 when calculating membrane potential, while Euler is more computationally efficient (de Alteriis, 2021). A combination of Euler and RK2 is commonly used to integrate membrane potentials. See Chou (2018).

[1] de Alteriis, G., & Oddo, C. M. (2021, May). Tradeoff between accuracy and computational cost of Euler and Runge Kutta ODE solvers for the Izhikevich spiking neuron model. In 2021 10th International IEEE/EMBS Conference on Neural Engineering (NER) (pp. 730-733). IEEE.

[2] Chou, T. S., Kashyap, H. J., Xing, J., Listopad, S., Rounds, E. L., Beyeler, M., ... & Krichmar, J. L. (2018, July). CARLsim 4: An open source library for large scale, biologically detailed spiking neural network simulation using heterogeneous clusters. In 2018 International joint conference on neural networks (IJCNN) (pp. 1-8). IEEE.

A few minor points in the Introduction section: 1st, “fast spiking interneurons” should be “fast-spiking interneurons.” “Non-fast spiking” should be “non-fast-spiking.” And use the abbreviation consistently in the latter text. 2. In the 2nd paragraph: .. updating, IS (not are) not well understood. 3rd, ..(ADP) with A time constant .. 4th, Last paragraph. Pyramidal cells EXCITE two types of interneurons, while nFS INs inhibit the (apical) dendritic compartment of pyramidal cells and FS INs inhibit the soma compartment.

Reply: Thank you! We rechecked the grammar and corrected those mistakes.

REVIEWER 2

In this study, authors constructed a spiking network model to investigate the underly circuitry mechanism of working memory task with sequentially or concurrently presented items. The study is overall interesting. However, I found the impact of the study is somewhat limited and the manuscript itself needs significant improvements, especially for figures part.

Reply: thanks for your comments.

1. In the first part of results, authors made several claims in several places, but they were not clear in the plots. For instances, “and FS almost do not discharge...”, “and form a localized oscillatory population activity (oscillatory activity bump)...” and “FS are suppressed after cue presentation...”. All of them is not clearly identifiable in plots. Theerfore, Figure 2 for this section needs revision for a better demonstration what authors wanted to claim. I suggest that authors should have some companying plots showing the average FR for each group as time evolved.

Reply: We apologies for the confusing descriptions.

As shown in Figure 2A, pyramidal cells showed relatively sparse and regular discharges in the absence of stimulation from 0-500 ms. At 500-700 ms, the stimulus elicited an intense firing in the cued direction. When the cue was withdrawn, this intense discharge in the preferred direction persisted and showed oscillatory activity throughout the 700-2000 ms delay period. We call this sustained oscillatory activity in the cued direction “oscillatory activity bump” and believe that it maintains information about the cue. Pyramidal cells in other directions were unaffected during and after the cue presentation and continued to fire sparsely and regularly. The inhibitory interneurons nFS (blue in Figure 2B) and FS (red in Figure 2B) showed opposite trends. In the pre-stimulus period (0-500 ms), the nFS discharged as sparsely and regularly as the pyramidal cell, whereas the FS barely discharged. Within 500-700 ms of stimulus presentation, the nFS stopped firing in the preferred direction, while the FS began to fire strongly and intensely in the preferred direction. This firing pattern continued throughout the 700-2000 ms delay period after stimulus withdrawal. The stimulus barely affected the non-preferred nFS and FS, and their spikes remained sparse or absent. Figure 4B provides additional information on the average membrane potential of the cells. It also gives examples of pyramidal cells, FS, and nFS in the preferred and non-preferred directions.

Hope everything is clear now.

2. For Figure 4C, I don’t see a clear drop in the total synaptic current for nFS after cue presents and delay period, as authors claimed in text.

Reply:

There was a relatively significant decrease in the total synaptic current of the NFS after the stimulus onset. Prior to the stimulus onset, the mean total synaptic current of the NFS was greater than 0, whereas after the stimulus presentation, the value was less than 0. We performed an independent samples t-test, which showed a significant result (Mpre=0.076, Maft=-0.241, t=100.96, p<0.001, Fig.6C).

3. In “WM load enhances the Gamma power during the delay period” section, when authors said “more cues elicit more distinct activity bumps with gamma oscillation”, is it because of the FR changes due to higher excitation brought by more cues?

Reply:

In our model, the increase in gamma power is likely due to more neurons involved in intensely firing oscillatory activity rather than a higher FR due to the increased excitation. After presenting one or three simultaneous cues, we compared the mean firing rates of 100 neurons in a preferred direction (from -4.39 to 4.39 degrees). We found that the firing rate in the three-cue condition was not higher (significantly lower) than in the one-cue condition (Mone=2.53, Mthree=2.17, t=21.72, p<0.001). In physiological experiments, the mechanism of gamma power increases with WM load may be more complex.

4. Regarding the last para of discussion, there was a recent modeling study by Feng et al., 2021 at 10th NER (International IEEE/EMBS Conference on Neural Engineering), which hypothesized one neocortical microcircuit can support both beta and gamma oscillations, but from different underlying mechanisms. This is very similar to what authors found here (Feng et al used two types of STP). Can authors discuss on it?

Reply:

Thanks for the suggestion. STP plays an essential role in the modulation of gamma and beta bursts. In the biological brain, there is short-term synaptic facilitation between pyramidal cells and nFS and short-term synaptic depression between pyramidal cells and FS. This short-term plasticity allows the brain to respond differently to afferents of different durations. Brief currents cause gamma oscillations, while prolonged currents cause beta oscillations. Based on this mechanism, Feng et al. developed a model in 2021 to reproduce these phenomena (Feng et al., 2021). In our research, we only briefly present the stimulus to the model, resulting in oscillations at the gamma frequency, and short-term synaptic facilitation between pyramidal cells and nFS helps control excitability in our model. The synapses equipped with STP can temporarily increase inhibition by increasing the effective excitation from the pyramidal cells to the nFS and expanding the range of parameters to keep excitation and inhibition in balance. Too much excitation can lead to the merging of different information (Wei et al,2012) or false memories (Edin et al,2008), while too much inhibition can prevent the network from maintaining information. Suppose the synapses from pyramidal cells to the nFS are fixed without STP; the network is prone to produce spurious bursts of activity due to too much excitation or to forget the information due to too much inhibition. At present, we have not considered the effects of prolonged stimulation. In future research, it would be interesting to investigate how the brain adapts and responds to prolonged stimulation.

5. Was there any particular reason for choosing number of 4098 pyramidal neurons, 512 FS and 512 nFS? And all the parameters authors chose were for some specific purposes or were they used for calibration of the network model biophysically?

Reply:

First, according to Marom and Shahaf (2002), the ratio of excitatory to inhibitory neurons in the brain is about 4:1. Considering only three cell types, the ratio of pyramidal neurons (4096) to inhibitory interneurons (FS+nFS, 1024) should approach 4:1. Second, when running the Fast Fourier Transform (FFT) algorithm, it is often desirable for the number of neurons to be a power of 2. The FFT algorithm recursively divides the input data into smaller subsets, performs Fourier transform computations on these subsets, and then combines the results in a particular way. If the number of neurons is a power of 2, it is well suited to the requirements of the FFT algorithm. The implementation of the model should be the same for other configurations of the number of neurons.

6. Authors should provide statistical tests where there were any comparisons.

Reply:

Thanks, we added statistical test results in the text. By the way, our model generated substantial data from multiple simulations with high power and narrow confidence intervals. We did not perform power and effect size tests because comparing these powers and confidence intervals directly with physiological standards would be unfair.

7. Minor: Please specify what band ranges are of interests in this study for Beta and Gamma in Abstract, Significance Statement and Introduction

Reply:

The range of interest is 10-30Hz for the beta oscillation and 30-100Hz for the gamma oscillation. This is now demonstrated in the abstract, significance statement and introduction.

8. Minor: “Beta” and “Gamma” should be lower case if they were not in the beginning of the sentences. STP and STF need be extended in the text.

Reply:

Thanks, these problems are fixed in the new manuscript.
